# Intraparenchymal Penicillin G Injection Promotes Wound Healing and Lowers POPF in Pigs After Pancreatic Surgery

**DOI:** 10.3390/biomedicines13030650

**Published:** 2025-03-06

**Authors:** Jung Hyun Park, Jae Hyun Han, Dosang Lee, Kee-Hwan Kim, Tae Ho Hong, Ok-Hee Kim, Sang-Jin Jeon, Ho Joong Choi, Say-June Kim

**Affiliations:** 1 Department of Surgery, Eunpyeong St. Mary’s Hospital, College of Medicine, The Catholic University of Korea, Seoul 03312, Republic of Korea; angle49@catholic.ac.kr; 2 Catholic Central Laboratory of Surgery, Institute of Biomedical Industry, College of Medicine, The Catholic University of Korea, Seoul 06591, Republic of Korea; dosangs@catholic.ac.kr (D.L.); keehwan@catholic.ac.kr (K.-H.K.); gshth@catholic.ac.kr (T.H.H.); ok6201@hanmail.net (O.-H.K.); chsach3492@naver.com (S.-J.J.); 3 Department of Surgery, St. Vincent Hospital, College of Medicine, The Catholic University of Korea, Suwon 16247, Republic of Korea; gelasius1@catholic.ac.kr; 4 Department of Surgery, Seoul St. Mary’s Hospital, College of Medicine, The Catholic University of Korea, Seoul 06591, Republic of Korea; 5 Department of Surgery, Uijeongbu St. Mary’s Hospital, College of Medicine, The Catholic University of Korea, Uijeongbu 11765, Republic of Korea; 6 Translational Research Team, Surginex Co., Ltd., Seoul 06591, Republic of Korea

**Keywords:** postoperative pancreatic fistula, penicillin G (PG), pancreatic fibrosis, pancreatic hardness, wound healing

## Abstract

**Background:** Postoperative pancreatic fistula (POPF) is a significant complication following pancreatic surgery, considerably influenced by the texture of the pancreatic tissue. This study aims to explore the potential of Penicillin G (PG) in reducing the severity of POPF in a porcine surgical model. **Study Design:** After performing distal pancreatectomy with pancreaticojejunostomy (PJ), pigs were administered either normal saline or varying concentrations of PG (0.75, 1.5, and 3.0 mM) at the PJ site. The study estimated POPF by measuring pancreatic hardness, tensile force, fibrosis, and amylase levels in Jackson-Pratt (JP) drain samples. **Results:** Intraparenchymal PG injection significantly increased pancreatic hardness and tensile force (*p* < 0.05) while upregulating profibrotic markers like MMP2 and TGF-β1, indicating enhanced fibrosis (*p* < 0.05). Importantly, these profibrotic changes reverted to baseline levels by POD 14, suggesting reversible fibrosis without lasting consequences. The 0.75 PG and 1.5 PG groups exhibited significantly lower JP amylase levels than the control group on both POD 3 and POD 4 (*p* < 0.05). Notably, the 0.75 PG group also demonstrated the highest survival rate compared to the 1.5 PG and NS groups (*p* < 0.05). **Conclusions:** The intrapancreatic PG injection could effectively reduce the severity of POPF by promoting wound healing through intensified fibrosis around the PJ site.

## 1. Introduction

Postoperative pancreatic fistula (POPF) is one of the most common and severe complications following pancreatic surgery. It is characterized by an abnormal communication between the pancreatic ductal system and another epithelial surface, leading to the leakage of enzyme-rich pancreatic fluid into the peritoneal cavity [1,2,3,4]. The incidence of POPF varies widely, reported as between 3% and 45% of pancreatic operations at high-volume centers [1,3,4,5,6]. However, clinically relevant POPF (grades B and C) occurs in approximately 11% of cases [1,2,3,4]. POPF is a major contributor to postoperative morbidity and mortality, accounting for up to 40% of all pancreatic surgery-related deaths [7,8,9,10]. The condition is also associated with longer hospital stays, increased healthcare costs, and reduced postoperative quality of life [11,12,13]. Several predictive markers and risk factors for POPF have been investigated to improve early detection and management strategies.

Measurement of POD 3 drain amylase is a well-established predictor of clinically relevant postoperative pancreatic fistula (CR-POPF). Studies have confirmed its strong predictive value, aiding early risk stratification. A POD3 drain amylase threshold of 500 IU/L is significantly associated with CR-POPF, with 83% sensitivity and 79% specificity [14]. A more refined cutoff of 1044 U/L demonstrated the highest accuracy (AUC: 0.843, sensitivity: 73%, specificity: 79%) [15]. Additionally, POD1 drain amylase may serve as an early indicator [14]. These findings confirm POD3 drain amylase as a reliable biomarker for CR-POPF, allowing risk-based drain management to optimize early intervention while facilitating safe, early drain removal [16].

A comprehensive meta-analysis of 27 studies, encompassing more than 24,000 patients, has unequivocally established that soft pancreatic texture is the most critical determinant of POPF following pancreaticoduodenectomy [17]. Meta-analyses and large-scale studies consistently demonstrate that a soft pancreas significantly increases the risk of POPF, with odds ratios ranging from approximately 3.5 to over 13 compared to a firm, fibrotic pancreas [18,19]. A landmark meta-analysis of 27 studies involving more than 24,000 patients identified soft pancreas as the strongest independent predictor of POPF, with a nearly four-fold increase in risk [17]. Similarly, the ISGPS validated a classification system based on pancreatic texture and duct size, confirming that a soft gland, especially in combination with a small duct, represents the highest-risk phenotype for POPF [18]. Across diverse cohorts, including single-center validations and prospective trials, pancreatic texture consistently emerges as the pivotal determinant of POPF risk, underscoring its central role in postoperative complications [19,20,21].

The most fundamental strategy to prevent POPF is to alter pancreatic texture from soft to firm or hard, as a softer pancreas is more prone to leakage due to its friability and poor suture-holding capacity [18]. Fibrosis plays a crucial role in achieving this transformation, as it is an essential process in wound healing that leads to the deposition of extracellular matrix (ECM) components, particularly collagen, which reinforces tissue integrity [22]. During fibrosis, pancreatic stellate cells (PSCs) and fibroblasts become activated in response to injury, undergoing transdifferentiation into myofibroblast-like cells that secrete large amounts of ECM proteins, including collagen types I and III [23]. This ECM accumulation increases tissue stiffness by creating a dense connective tissue network that encapsulates parenchymal cells, mechanically supporting and stabilizing the pancreatic architecture [24]. The deposition of fibrillar collagen physically restricts pancreatic tissue deformation, reducing its compliance and making it less susceptible to mechanical disruption, thereby preventing POPF [25].

In our previous study, we screened various compounds to identify potential profibrotic materials for the prevention of POPF and discovered that Penicillin G (PG) exhibits significant profibrogenic properties [26]. Using a mouse model, we showed that intrapancreatic injection of PG induces reversible fibrosis by activating PSCs through transforming growth factor-β (TGF-β) signaling. Building on these findings, the present study aims to evaluate the clinical relevance of PG-induced fibrosis in a porcine model undergoing major pancreatic surgery, including pancreatectomy and PJ. To achieve this, we measured amylase levels in the pancreatic juice collected from Jackson-Pratt (JP) drains, a standard clinical indicator of POPF in human patients. By assessing whether intrapancreatic injection of PG can reduce JP amylase levels, we seek to determine its potential efficacy as a therapeutic strategy for preventing POPF in a surgical setting.

## 2. Materials and Methods

### 2.1. Animals & Study Design

**Figure 1:**
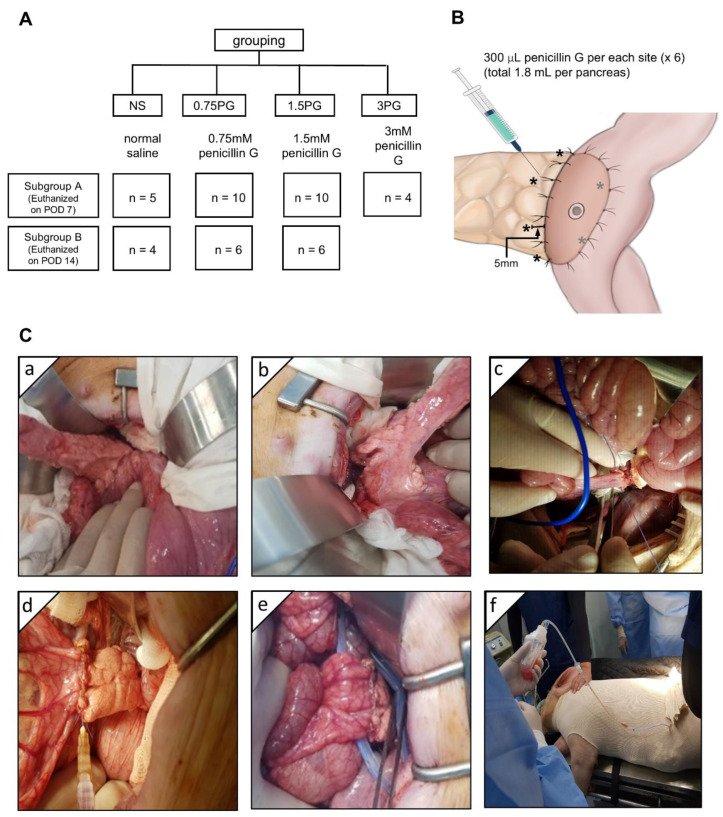
Experimental design and procedure. (**A**) Study design. (**B**) Intrapancreatic injection site (*). After pancreaticojejunostomy (PJ), 300 μL (total 1.8 mL) of normal saline (NS), 0.75 mM PG, or 1.5 mM PG was injected at six locations around the circumference of the pancreas located within 1 cm from the PJ site. (**C**) Operative illustration. After laparotomy, the pancreas was identified and exposed (**a**). The pancreatic tail was divided at about 3 cm from the back (**b**), and then end-to-side PJ was performed with the proximal jejunum (**c**). The animals in the NS, 0.75 PG, and 1.5 PG groups received, respectively, NS, 0.75 mM PG, and 1.5 mM PG to the periphery of the pancreas where PJ was established (**d**). Both ends of the JP drain tips were placed adjacent to the PJ site, anteriorly and posteriorly, respectively (**e**). The pig was clothed for the prevention of dislocation and for fixation of the JP drain (**f**). Abbreviations: 0.75 PG, intrapancreatic injection of 0.75 mM PG; 1.5 PG, intrapancreatic injection of 1.5 mM PG; NS, intrapancreatic injection of 1.8 mL NS.

The animal study was conducted in accordance with the guidelines of the Institute for Laboratory Animal Research, Korea (IRB No: CRONEX-IACUC: 202102001). A total of 42 healthy adult female Yorkshire, Berkshire, Duroc (YBD) pigs (Munhwa Farm, Pyeongtaek, Gyeonggi, Republic of Korea) were used in this study (Figure 1A). The animals were acclimatized for two weeks in an environment with controlled temperature (24 ± 2 °C), humidity (50 ± 10%), light-dark cycle (12-h) and ventilation (10–15 times/h). The pigs were fasted for 12 h before the surgery and premedicated with 20 μ/kg intramuscular tiletamine/zolazepam. Respiratory anesthesia was performed using a gas mixture of isoflurane and oxygen. The animals were divided into four groups: normal saline (NS), 0.75 PG, 1.5 PG, and 3 PG. Each group was further divided into two subgroups based on the timing of sample collection: one subgroup on postoperative day (POD) 7 and the other subgroup on POD 14. Before the surgery, the injection site for the intrapancreatic injection was determined, and 300 μL (total 1.8 mL) of material was planned to be injected at six locations around the circumference of the pancreas located within 1 cm from the pancreaticojejunostomy (PJ) site (Figure 1B). After laparotomy, the pancreas was identified and exposed (Figure 1C(a)). The pancreatic tail was divided at approximately 6 cm from the back (Figure 1C(b)), and then end-to-side PJ was performed between the pancreatic stump and proximal jejunum (Figure 1C(c)). The animals in the NS, 0.75 PG, and 1.5 PG groups received, respectively, intraparenchymal injection of NS, 0.75 mM PG, and 1.5 mM PG (Figure 1C(d)). Both tips of the JP drain (Sewoonmedical, Seoul, Korea) lines were placed anteriorly and posteriorly to the PJ anastomosis (Figure 1C(e)), and the other sides of the tips were pulled out of the abdominal cavity and fixed. After abdominal wall closure in a layer-by-layer fashion, the pig was clothed for the stable maintenance of the JP drain bag, which was fixed in a position where the pig’s head, legs, and tail could not reach (Figure 1C(f)).

### 2.2. Measurement of Hardness and Tensile Strength of Pig Pancreas

**Figure 2:**
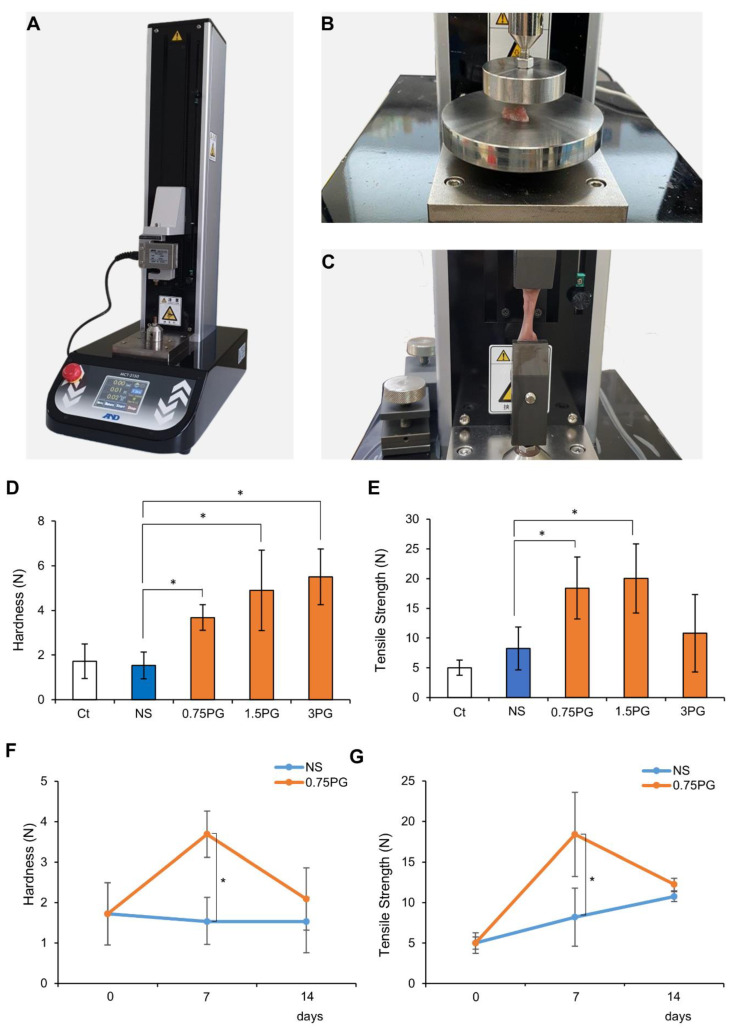
PG effects on pancreatic hardness and tensile force. (**A**) Tensile strength tester used to measure pancreatic hardness and tensile strength following injections of NS or PG. A mechanical device was employed to assess the physical properties of pancreatic tissue. (**B**) Measurement of pancreatic hardness after NS or PG injection. Hardness was quantified to evaluate the effects of PG on pancreatic tissue stiffness. (**C**) Measurement of pancreatic tensile strength after NS or PG injection. Tensile strength was measured to determine the structural integrity of the pancreas after PG injection. (**D**) Measurement of pancreatic hardness after NS or PG injection. Pancreatic hardness on POD 7 was significantly increased in all PG-treated groups (0.75 PG, 1.5 PG, and 3 PG) compared to NS. (**E**) Comparison of pancreatic tensile strength of NS, 0.75 PG, 1.5 PG, and 3 PG on POD 7. Tensile strength on POD 7 was significantly increased in the 0.75 PG and 1.5 PG groups compared to NS, with no significant difference between them, while the 3 PG group showed no significant increase. (**F**) Comparison of pancreatic hardness between NS and 0.75 PG groups at POD 0, 7, and 14. Pancreatic hardness in the 0.75 PG group was significantly higher than NS on POD 7 but returned to a level similar to NS by POD 14. (**G**) Comparison of pancreatic tensile strength between NS and 0.75 PG groups at POD 0, 7, and 14. Tensile strength in the 0.75 PG group was significantly higher than NS on POD 7 but showed no significant difference between groups by POD 14. Values are presented as mean ± standard deviation of three independent experiments. * *p* < 0.05. Abbreviations: 0.75 PG, intrapancreatic injection of 0.75 mM penicillin G; 1.5 PG, intrapancreatic injection of 1.5 mM penicillin G; 3 PG, intrapancreatic injection of 3 mM penicillin G; NS, normal saline; POD, postoperative day.

A tensile strength tester (MCT-2150; A&D Korea Co., Seoul, Republic of Korea) was utilized to measure the hardness and tensile force of the pig pancreas at the site of control and tested materials injection. The process of measuring pancreatic hardness involves placing the durometer on the pancreas and operating the machine (Figure 2A). Afterward, the tissue was fixed at both ends of the machine strokes, and tensile strength was measured at a speed of 50 mm/min (Figure 2B,C).

### 2.3. Western Blotting Analysis

Pancreatic tissues were washed with phosphate buffer solution (PBS) and lysed in an EzRIPA Lysis kit (ATTO Corporation, Tokyo, Japan). The protein concentration was determined using Bradford reagent (Bio-Rad, Hercules, CA, USA). The proteins were visualized by Western blot analysis using primary antibodies (1:1000 dilution) at 4 °C overnight, and then with HRP-conjugated secondary antibodies (1:2000 dilution) for 1 h at 24 °C. The primary antibodies used for this study were transforming growth factor-β1 (TGF-β1), metalloproteinase-2 (MMP2), and β-actin (Cell Signaling, Beverly, MA, USA). The specific immune complexes were detected using Western Blotting Plus Chemiluminescence Reagent (Millipore, Billerica, MA, USA).

### 2.4. Masson’s Trichrome Staining and Immunohistochemistry

Masson’s trichrome staining was performed using a Masson’s trichrome staining kit (Polysciences, Warrington, PA, USA), according to the manufacturer’s protocol. For immunohistochemical analysis, formalin-fixed, paraffin-embedded tissue sections were deparaffinized, rehydrated in an ethanol series, and subjected to epitope retrieval using standard procedures. The primary antibodies used for this study were TGF-β1 and MMP2 (Cell Signaling Technology, Danvers, MA, USA). The samples were then examined under a laser-scanning microscope (Eclipse TE300; Nikon, Tokyo, Japan) to analyze the expression of these antibodies.

### 2.5. Enzyme-Linked Immunosorbent Assay (ELISA)

Serum samples were obtained from the heart, and the serum concentrations of porcine interleukin-6 (IL-6), tumor necrosis factor-α (TNF-α), and insulin were measured using sandwich enzyme-linked immunosorbent assay (ELISA; eBioscience Inc., San Diego, CA, USA and Biolegend Inc., San Diego, CA, USA, respectively) according to the manufacturer’s instructions.

### 2.6. Statistical Analysis

All data were analyzed using SPSS 11.0 software (SPSS, Chicago, IL, USA) and are presented as the mean ± standard deviation (SD). The Mann-Whitney U-test was used for the comparison of two groups, and the Kruskal-Wallis test was used for the comparison of three or more groups. Probability (*p*) values of less than 0.05 were considered statistically significant.

## 3. Results

### 3.1. PG Effects on Pancreatic Hardness and Tensile Force

A tensile strength tester (Figure 2A) was employed to quantify the hardness (Figure 2B) and tensile strength (Figure 2C) of pancreatic tissue following injections of NS or PG on POD 7. First, pancreatic hardness and tensile strength were assessed on POD 7 in each group. In the comparison of pancreatic hardness, all PG-treated groups (0.75 PG, 1.5 PG, and 3 PG) exhibited a significant increase compared to the NS group (Figure 2D). Among them, the 1.5 PG and 3 PG groups showed the highest increase, with no significant difference observed between these two groups. In the comparison of tensile strength, the 0.75 PG and 1.5 PG groups exhibited the most significant increase, with no significant difference between them (Figure 2E). However, in the 3 PG group, tensile strength was not significantly increased compared to the NS group. Subsequently, pancreatic hardness and tensile strength were compared between the 0.75 PG and NS groups across different PODs (POD 0, 7, and 14). For hardness, the 0.75 PG group exhibited a significant increase compared to the NS group on POD 7. By POD 14, although hardness appeared higher in the 0.75 PG group than in the NS group, this difference was not statistically significant (Figure 2F). For tensile strength, the 0.75 PG group showed a significant increase compared to the NS group on POD 7. However, by POD 14, the tensile strength in both groups had decreased to similar levels, and the difference was no longer statistically significant (Figure 2G). These findings suggest that pancreatic hardness and tensile strength, which reflect fibrosis, were significantly increased by 0.75 PG injection compared to NS on POD 7. However, by POD 14, this significant increase was no longer observed, indicating that the effect of 0.75 PG on enhancing pancreatic mechanical properties does not persist beyond POD 14.

### 3.2. PG Effects on Pancreatic Fibrosis and Tissue Remodeling

**Figure 3:**
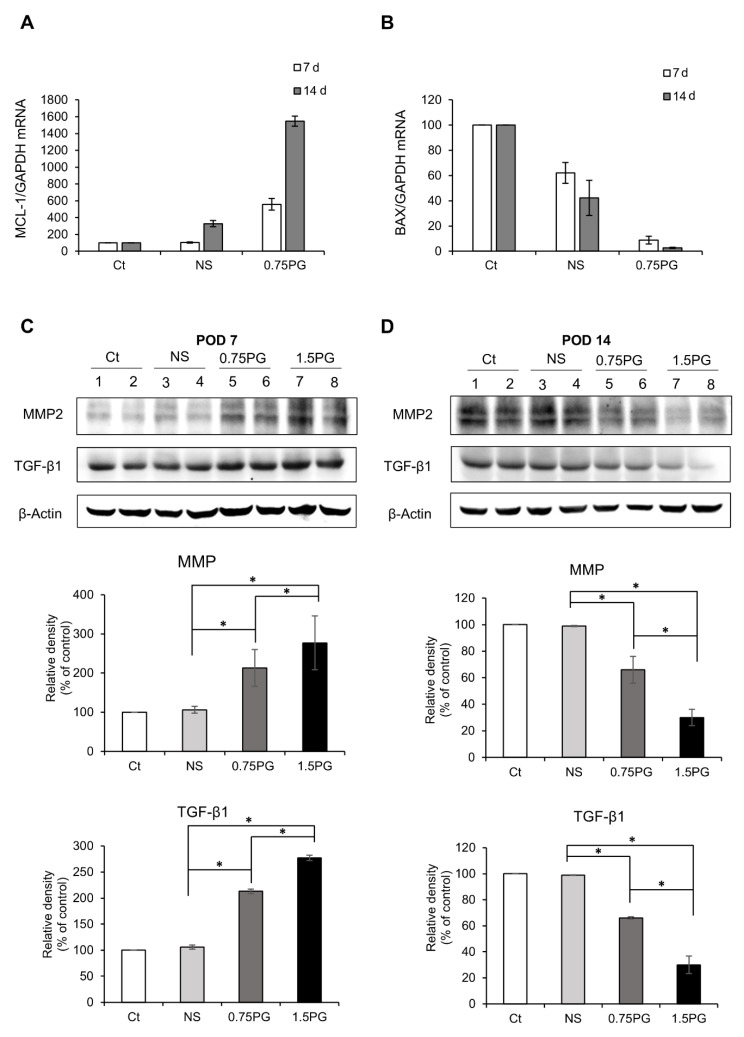
Analysis of apoptosis and fibrosis in the porcine pancreas following intrapancreatic PG injection. Real-time PCR analysis of pro-apoptotic marker BAX (**A**) and anti-apoptotic marker Mcl-1 (**B**) expression in pig pancreatic tissues injected with various materials. Comparing the 0.75 PG group to the control and NS groups, a significant decrease in the pro-apoptotic marker BAX expression was observed, along with an increase in the expression of the anti-apoptotic marker Mcl-1. (**C**) Western blotting analysis on POD 7 showing the expression of the profibrotic markers MMP2 and TGF-β1 in the pancreatic tissues in each group. The 1.5 PG group showed the highest expression of MMP2 and TGF- β1 (*p* < 0.05). (**D**) Western blotting analysis on POD 14 showing the expression of the profibrotic markers MMP2 and TGF- β1 in the pancreatic tissues in each group. The expression levels of the fibrotic proteins MMP2 and TGF-β1 proteins—which had been increased on POD 7—were normalized. Values are presented as mean ± standard deviation of three independent experiments. * *p* < 0.05. Abbreviations: 1.5 PG, intrapancreatic injection of 1.5 mM PG; 0.75 PG, intrapancreatic injection of 0.75 mM PG; Ct, control group; MMP-2, metalloproteinases-2; NS, intrapancreatic injection of 1.8 mL NS; TGF-β1, transforming growth factor-β1.

To evaluate apoptosis in pig pancreatic tissues injected with various materials, we assessed the mRNA expression of the pro-apoptotic marker BAX and the anti-apoptotic marker Mcl-1 using real-time PCR. When comparing the 0.75 PG group to the Control and NS groups, we observed a significant decrease in the pro-apoptotic marker BAX expression (*p* < 0.05) (Figure 3A), along with an increase in the expression of the anti-apoptotic marker Mcl-1 (*p* < 0.05) (Figure 3B). This pattern became more pronounced at post-injection day 14.

Subsequently, to investigate the degree of pancreatic fibrosis, the expression levels of fibrosis-related proteins were analyzed using Western blotting on POD 7 and POD 14 in tissues obtained from the pancreatic stump where the injection of materials was given. The levels of the fibrotic markers, MMP2 and TGF-β1, were significantly higher in both the NS and 0.75 PG groups on POD 7 than in the control group (*p* < 0.05). Moreover, the expression levels of both markers were significantly higher in the 0.75 PG and 1.5 PG groups than in the NS group (*p* < 0.05) (Figure 3C). However, by POD 14, the increased expression levels of both markers in both the 0.75 PG and 1.5 PG groups had normalized (Figure 3D).

**Figure 4:**
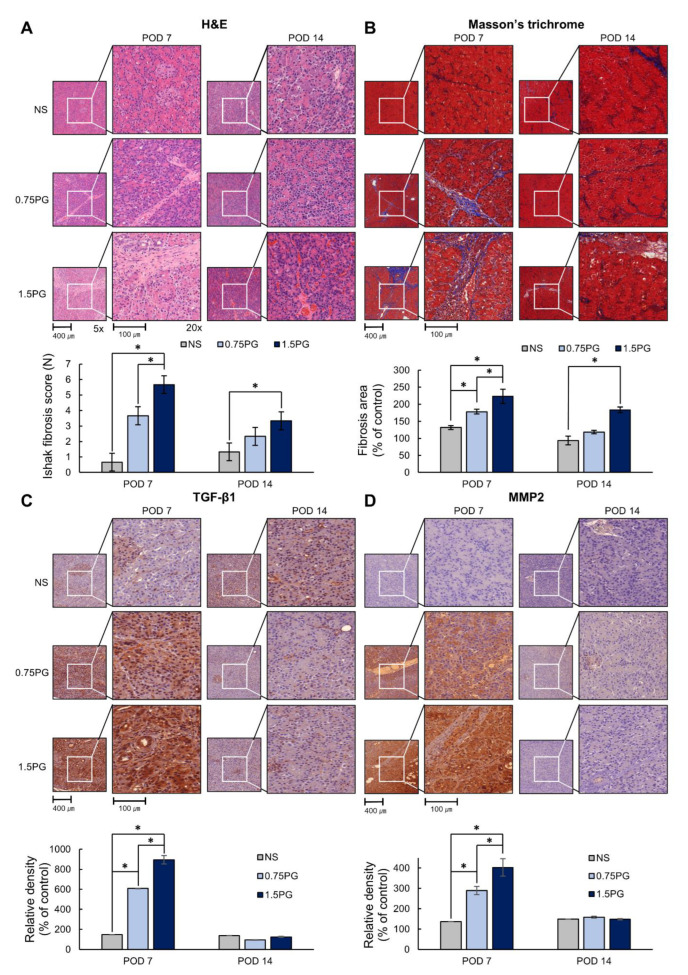
PG effects on pancreatic histological changes and fibrosis markers. (**A**) Hematoxylin and eosin staining of pig pancreas on POD 7 and 14. (**B**) Masson’s trichrome staining of pig pancreas on POD 7 and 14. (**C**) TGF-β1 immunohistochemistry in pig pancreas on POD 7 and 14. (**D**) MMP2 immunohistochemistry in pig pancreas on POD 7 and 14. These results indicated that the markers of fibrosis were significantly increased in the 0.75 PG and 1.5 PG groups compared to those in the NS group on POD 7. However, on POD 14, most of the increased markers in these groups were markedly reduced. Values are presented as mean ± standard deviation of three independent experiments. * *p* < 0.05. Abbreviations: 0.75 PG, intrapancreatic injection of 0.75 mM PG; 1.5 PG, intrapancreatic injection of 1.5 mM PG; IHC, immunohistochemistry; MMP-2, metalloproteinases-2; NS, intrapancreatic injection of 1.8 mL NS; TGF-β1, transforming growth factor-β1.

Subsequently, the degree of fibrosis in pancreatic tissues was determined by various stains, including hematoxylin and eosin (H&E), Masson’s trichrome, and TGF-β1 and MMP1 immunohistochemical (IHC) stains. H&E staining showed that the Ishak fibrosis score was significantly higher in both the 0.75 PG and 1.5 PG groups than in the NS group on POD 7 (*p* < 0.05) (Figure 4A). Additionally, the fibrosis score was significantly higher in the 1.5 PG group than in the 0.75 PG group (*p* < 0.05). On POD 14, the increased fibrosis score in both the 0.75 PG and 1.5 PG groups decreased, although the fibrosis score was still significantly higher in the 1.5 PG group than in the NS group (*p* < 0.05). Masson’s trichrome stain demonstrated a similar pattern to the H&E stain (Figure 4B). Furthermore, TGF-β1 and MMP2 IHC staining showed a significant increase in the percentage of immunoreactive areas in both the 0.75 PG and 1.5 PG groups compared to the NS group on POD 7 (*p* < 0.05) (Figure 4C,D). The immunoreactive areas of the 1.5 PG group were significantly larger than those of the 0.75 PG group (*p* < 0.05). On POD 14, the increased immunoreactive areas in both the 0.75 PG and 1.5 PG groups decreased, approaching the level of the NS group.

### 3.3. PG Effects on Hepatic, Renal, Systemic Inflammatory, and Pancreatic Endocrine Function

**Figure 5:**
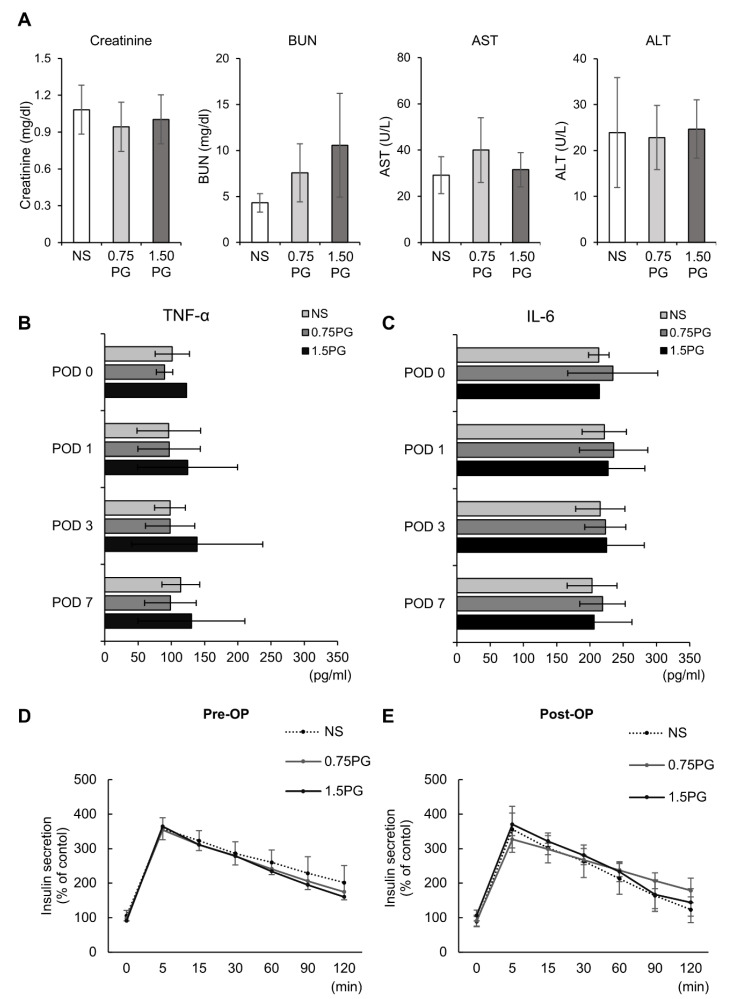
Evaluation of hepatic, renal, systemic inflammatory, and pancreatic endocrine function following intrapancreatic PG injection. (**A**) kidney function (creatinine, BUN) and liver function (AST, ALT) markers were assessed across experimental groups (NS, 0.75 PG, and 1.5 PG), showing no significant differences between groups. (**B**) Comparison of serum concentration of TNF-α in each group over time. (**C**) Comparison of serum concentration of IL-6 in each group over time. (**D**) Pre-injection comparison of serum levels of insulin at certain time intervals by ELISA after glucose infusion in each group. (**E**) Post-injection comparison of serum levels of insulin at certain time intervals by ELISA after glucose infusion in each group. Values are presented as mean ± standard deviation of three independent experiments. Abbreviations: 0.75 PG, intrapancreatic injection of 0.75 mM PG; 1.5 PG, intrapancreatic injection of 1.5 mM PG; NS, intrapancreatic injection of 1.8 mL NS; TGF-β1, transforming growth factor-β1.

To comprehensively assess the systemic effects of PG, renal function (creatinine, BUN), hepatic function (AST, ALT), systemic inflammatory response (TNF-α, IL-6), and pancreatic endocrine function (insulin secretion) were evaluated across experimental groups. First, renal and hepatic function tests showed no significant differences in creatinine, BUN, AST or ALT levels between groups, indicating that PG injection did not impair liver or kidney function (Figure 5A). Next, to determine whether intrapancreatic PG injection triggers a systemic inflammatory response, we measured serum levels of TNF-α and IL-6, two well-established systemic inflammatory markers. No significant differences in TNF-α and IL-6 levels were observed among the groups, suggesting that PG administration did not provoke systemic inflammation and may be safe for clinical use (Figure 5B). Finally, to evaluate the impact of PG on pancreatic endocrine function, serum insulin levels were measured at multiple time points following the administration of 25% glucose (0.5 g/kg) into the ear vein. The comparison of insulin secretion patterns between pre- and post-treatment samples revealed no significant differences among the groups, indicating that PG does not impair pancreatic endocrine function (Figure 5C,D).

### 3.4. PG Effects on the Development of POPF and Pig Survival

**Figure 6:**
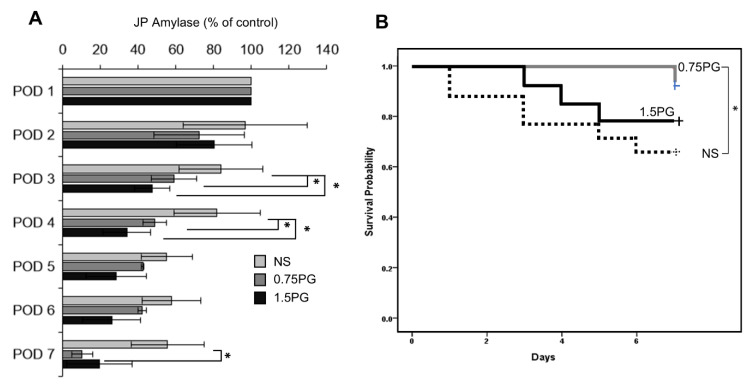
PG effects on the development of POPF and pig survival. (**A**) Analysis of JP amylase levels in the experimental groups over a seven-day period. On POD 3, JP amylase levels were significantly lower in the 0.75 PG and 1.5 groups than in the NS group (*p* < 0.05). Interestingly, on POD 7, JP amylase levels were also significantly lower in the 0.75 PG than in the NS group (*p* < 0.05). Values are presented as mean ± standard deviation of three independent experiments. (**B**) Kaplan-Meier survival analysis of the 0.75 PG, 1.5 PG, and NS groups. The 0.75 PG group exhibited the highest survival rate, with a significant difference in survival rates observed between the 1.5 PG group and the NS group. * *p* < 0.05. Abbreviations: 0.75 PG, the group with intrapancreatic injection of 0.75 mM PG; 1.5 PG, the group with 1.5 mM PG; NS, the group with 1.8 mL NS.

To assess whether PG is a potential intervention for reducing the incidence and severity of POPF, JP amylase levels were analyzed in the experimental groups over a period of seven days, including POD 3, 4, and 7 (Figure 6A). The results demonstrated a significant reduction in JP amylase levels on POD 3 and 4 in both the 0.75 PG and 1.5 PG groups compared to the NS group (*p* < 0.05), indicating a reduced incidence of POPF in these groups. Moreover, there was no significant difference in JP amylase levels between the 0.75 PG and 1.5 PG groups, suggesting that a lower dose of PG may be sufficient to achieve the desired effect. Interestingly, on POD 7, JP amylase levels were significantly lower in the 0.75 PG group than in the NS group (*p* < 0.05), indicating that the effect of PG on reducing the incidence of POPF persisted beyond the initial postoperative period. These findings suggest that intrapancreatic injection of PG could be a promising strategy for reducing the incidence and severity of POPF, thus improving postoperative outcomes in pancreatic surgery. In Kaplan-Meier survival analysis, among the 0.75 PG, 1.5 PG, and NS groups, the 0.75 PG group exhibited the highest survival rate, while there was a significant difference in survival rates between the 1.5 PG group and the NS group (Ps < 0.05) (Figure 6B).

## 4. Discussion

This study investigated the effects of intrapancreatic PG injection on pancreatic fibrosis and POPF prevention in a porcine model of pancreatic resection and PJ. PG was directly injected into the pancreatic parenchyma, and its effects were assessed through three primary evaluations: (1) blood tests, including inflammatory markers such as TNF-α and IL-6, (2) pancreatic tissue analysis, including histological staining and Western blotting for fibrosis-related markers (TGF-β1 and MMP2), and (3) direct POPF assessment, measured using JP drain amylase levels, following the same manner as in human patients. The results demonstrated that PG injection increased pancreatic hardness and upregulated fibrosis-related markers, such as TGF-β1 and MMP2, promoting a fibrotic response. Notably, while the 1.5 mM PG group exhibited a stronger and more prolonged fibrotic reaction, fibrosis in the 0.75 mM PG group was reversible, returning to baseline levels by POD 14. Additionally, the 0.75 mM PG group significantly reduced JP amylase levels, indicating a decrease in POPF severity. These findings suggest that 0.75 mM PG effectively induces transient fibrosis while also mitigating POPF, making it the optimal concentration for achieving both efficacy and safety in this surgical model.

A soft pancreatic texture is a well-established risk factor for POPF due to technical challenges during anastomosis, where excessive suture tightening can cause compression ischemia, leading to tissue necrosis and pancreatic juice leakage [27,28]. Conversely, loosening the anastomosis can create a dead space, further increasing the risk of POPF [28]. Additionally, higher exocrine activity, a smaller pancreatic duct diameter (<3 mm), and numerous small ductal branches contribute to proteolytic tissue breakdown, fistula formation, and delayed healing [26,27,29,30,31]. These factors collectively increase the likelihood of POPF in patients with a soft pancreas.

**Figure 7:**
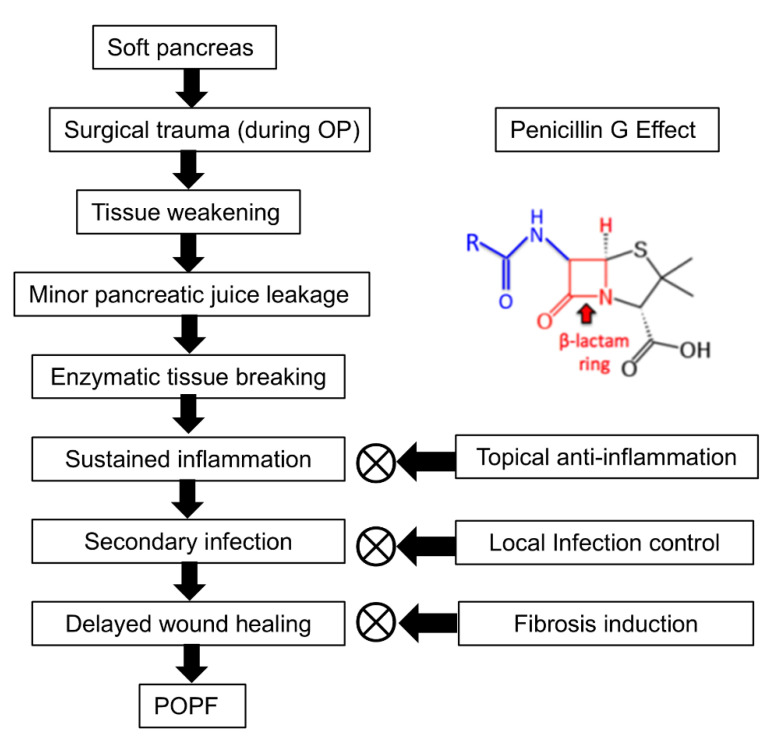
Pathogenesis of POPF and the proposed role of PG. POPF development begins with a soft pancreas, which undergoes surgical trauma and subsequent tissue weakening, making it susceptible to minor pancreatic juice leakage. This leakage leads to enzymatic tissue breakdown, sustained inflammation, secondary infection, and delayed wound healing, ultimately resulting in POPF formation. PG is suggested to mitigate POPF progression by potentially reducing inflammation, controlling infection, and promoting fibrosis. By modulating cytokine activity, limiting bacterial overgrowth, and enhancing ECM synthesis, PG may help stabilize pancreatic tissue and improve wound healing around the anastomotic site. However, the precise molecular mechanisms underlying these effects require further investigation.

PG is presumed to mitigate POPF through three primary mechanisms: topical anti-inflammation, local infection control, and fibrosis induction (Figure 7). First, PG exhibits anti-inflammatory effects by reducing neutrophil infiltration and suppressing pro-inflammatory cytokines such as IL-6 and TNF-α. A study demonstrated that the combination of sodium houttuyfonate and PG significantly downregulated inflammatory responses and enhanced wound healing in a methicillin-resistant Staphylococcus aureus (MRSA)-infected rat model [32]. In this study, sodium houttuyfonate and PG were topically applied to the wound site, leading to a marked reduction in neutrophil infiltration and decreased levels of IL-6 and TNF-α in the blood. Wound healing was assessed through measurements of wound area reduction and histological analyses, which showed increased collagen fiber deposition, indicating enhanced fibroblast activity. Second, as a β-lactam antibiotic, PG exerts its antibacterial effect by disrupting bacterial cell wall synthesis. The β-lactam ring in PG targets penicillin-binding proteins, crucial enzymes in the synthesis of peptidoglycan, an essential component of the bacterial cell wall. This inhibition leads to cell lysis and death in susceptible bacteria. Topical administration of PG can achieve higher local antibiotic concentrations at the surgical site compared to systemic administration. For instance, locally applied vancomycin in spine surgery has been shown to achieve tissue concentrations up to 1000 times the minimum inhibitory concentration (MIC) for common pathogens, persisting for POD 2–3, with relatively low systemic concentrations [33].

In addition to its anti-inflammatory and antibacterial effects, our previous study suggested that PG may play a key role in fibrosis induction by interacting with the TGF-β signaling pathway, leading to fibroblast proliferation and ECM deposition [26]. Specifically, intrapancreatic injection of PG was associated with the activation of human PSCs, significantly upregulating fibrosis-related markers, including TGF-β1, p-SMAD, COL1A1, MMP2, and TIMP1. This profibrotic response appeared to increase pancreatic hardness, reinforcing the pancreatic tissue structure. The potential link between PG and TGF-β receptor activation was further examined through inhibition studies, where treatment with a TGF-β1 receptor inhibitor significantly reduced the increase in pancreatic hardness induced by PG. This controlled induction of fibrosis may help stabilize the pancreatic tissue, support the anastomotic site, and reduce pancreatic juice leakage, ultimately suggesting a role in the prevention of POPF. However, for patients with PG allergy, alternative β-lactam antibiotics that share a similar core structure, such as cefazolin (a first-generation cephalosporin) or meropenem (a carbapenem), may be considered as substitutes [34]. These alternatives retain β-lactam activity while exhibiting broader or modified antibacterial spectra. Nevertheless, further studies are necessary to clarify the precise molecular mechanisms underlying PG-induced fibrosis and to evaluate its clinical applicability in wound healing.

Currently, various preventive strategies are used in combination to minimize its occurrence, though none have demonstrated clearly superior efficacy. Given its role in promoting wound healing and reducing bacterial load, PG may provide additional benefits when combined with existing preventive measures, potentially leading to a synergistic effect in POPF reduction. Among the widely studied approaches, somatostatin analogs have been shown to suppress pancreatic exocrine secretion, thereby lowering the enzymatic load at the anastomotic site and reducing the risk of leakage [35]. Additionally, intraoperative reinforcement techniques, including the use of local agents such as fibrin glue or Surgicel, as well as mechanical support materials like Vicryl mesh (polyglactin 910) or polyglycolic acid (PGA) mesh, have been utilized to strengthen the anastomosis and decrease POPF incidence [36,37]. Considering these existing strategies, the combination of PG with systemic therapies such as somatostatin analogs and intraoperative reinforcement materials could be an effective approach in further reducing POPF rates. Future studies should focus on evaluating the efficacy of this combined approach and determining the optimal treatment regimen for maximizing POPF prevention.

While this study primarily focuses on the prevention and treatment of POPF through intraparenchymal PG injection, its potential applications may extend to other types of soft tissue fistulas, such as anorectal or vesicovaginal fistulas, due to its antimicrobial properties and wound-healing effects. However, its direct clinical applicability requires further validation. Although the fibrotic response induced by PG was found to be reversible, its long-term impact on pancreatic function remains unclear and warrants further investigation. Additionally, the efficacy of PG in the presence of antibiotic-resistant bacteria, particularly Enterobacter spp. and other pathogens within the complex biliary microbiome, has not been fully assessed. Since PG’s antibacterial activity may be limited against resistant strains, its anti-inflammatory and fibrosis-inducing effects could play a more significant role in its therapeutic efficacy. Future research should prioritize clinical trials, long-term safety evaluations, and comparative studies with existing prophylactic agents to optimize POPF prevention while ensuring broad antimicrobial efficacy. Additionally, further studies are needed to determine the potential for combination therapies, such as the concurrent use of PG with agents targeting resistant bacterial strains, and whether PG could be effectively applied to other soft tissue fistulas beyond POPF.

## 5. Conclusions

In conclusion, intrapancreatic injection of PG significantly decreased the severity of POPF in a pig model undergoing pancreaticojejunostomy, likely due to increased pancreatic hardness and resulting tensile strength. The potential mechanism of this effect is the reinforcement of the fibrosis step of the wound healing process, as evidenced by significantly increased expression of pro-fibrotic proteins in the pancreatic tissue on the seventh day of injection. Furthermore, the enhanced fibrosis caused by 0.75–1.5 mM PG was reversible and did not result in long-term pancreatic damage. Importantly, the safety of PG was confirmed by the absence or minimal induction of systemic inflammatory responses and impairment of pancreatic endocrine function. Future clinical trials are needed to further determine the efficacy and toxicity of PG in patients undergoing pancreatic surgery.

## Data Availability

The datasets generated and/or analyzed during the current study are available from the corresponding author upon reasonable request.
